# Bafetinib (INNO-406) reverses multidrug resistance by inhibiting the efflux function of ABCB1 and ABCG2 transporters

**DOI:** 10.1038/srep25694

**Published:** 2016-05-09

**Authors:** Yun-Kai Zhang, Guan-Nan Zhang, Yi-Jun Wang, Bhargav A. Patel, Tanaji T. Talele, Dong-Hua Yang, Zhe-Sheng Chen

**Affiliations:** 1 Department of Pharmaceutical Sciences, College of Pharmacy and Health Sciences, St. John’s University, Queens, NY 11439, USA

## Abstract

ATP-Binding Cassette transporters are involved in the efflux of xenobiotic compounds and are responsible for decreasing drug accumulation in multidrug resistant (MDR) cells. Discovered by structure-based virtual screening algorithms, bafetinib, a Bcr-Abl/Lyn tyrosine kinase inhibitor, was found to have inhibitory effects on both ABCB1- and ABCG2-mediated MDR in this *in-vitro* investigation. Bafetinib significantly sensitized ABCB1 and ABCG2 overexpressing MDR cells to their anticancer substrates and increased the intracellular accumulation of anticancer drugs, particularly doxorubicin and [^3^H]-paclitaxel in ABCB1 overexpressing cells; mitoxantrone and [^3^H]-mitoxantrone in ABCG2 overexpressing cells, respectively. Bafetinib stimulated ABCB1 ATPase activities while inhibited ABCG2 ATPase activities. There were no significant changes in the expression level or the subcellular distribution of ABCB1 and ABCG2 in the cells exposed to 3 μM of bafetinib. Overall, our study indicated that bafetinib reversed ABCB1- and ABCG2-mediated MDR by blocking the drug efflux function of these transporters. These findings might be useful in developing combination therapy for MDR cancer treatment.

Cancer cells may involve several mechanisms of drug resistance such as efflux transporters, apoptosis regulation, autophagy regulation, DNA repair, and epigenetic regulation to become multi-drug resistance (MDR)^1^. Among these factors, the most prominent one is the increased efflux of chemotherapeutic drug mediated by transmembrane transporters. ATP-binding cassette (ABC) transporters present on plasma membranes are the superfamily of 49 members. The energy derived from ATP hydrolysis drives the transport of various endogenous ligands and exogenous drugs^2^. ABC transporters share several common structural features including transmembrane domains (TMDs) for ligand recognition and transport, as well as nucleotide-binding domains (NBDs) for ATP binding and hydrolysis at cytoplasmic site^3^. It is well established that these ABC transporters, particularly the ABC transporter subfamily B member 1 (ABCB1) and –subfamily G member 2 (ABCG2), play an important role in inducing MDR in cancer cells^4^,^5^. Overexpressions of ABCB1 and ABCG2 have been shown to produce MDR in various kinds of cancers, such as breast, colon, lung, ovarian cancers and melanomas^6^,^7^,^8^. Substrates of ABCB1 included anthracyclines, vinca alkaloid, taxanes, epipodophyllotoxins and so on, while ABCG2 was known to transport organic anion conjugates, nucleoside analogues, anthracyclines, methotrexate and flavopiridols^9^. Hence, it is essential to develop inhibitors of these transporters in order to overcome MDR and retrieve the effectiveness of conventional anticancer drugs. Moreover, since ABCB1 and ABCG2 can both be expressed in MDR cancer cells^10^, it is more favorable to develop an inhibitor which target at both ABCB1 and ABCG2.

Recently, a number of small molecule kinase inhibitors have been found to interact with ABC transporters^3^,^11^. These inhibitors were usually originally designed for other targets in cell-signal network and were found to be active towards several ABC transporters. These inhibitors were either clinically approved or under evaluation in clinical trials, such as ibrutinib, icotinib and nilotinib^12^,^13^,^14^. These inhibitors provided a fertile ground for the discovery of new ABC transporter inhibitors. Computational models constitute a fast and low-cost alternative to detect potential active compounds. Several pharmacophoric based or quantitative structure activity relationship (QSAR) based *in silico* studies on ABCB1 and ABCG2 in recent years have successfully discovered common features for ABC transporter binding^15^,^16^,^17^. On the other hand, a large hydrophobic drug binding pocket of ABCB1 inside the TMD was previous illustrated through co-crystallization of mice ABCB1 and its bound inhibitor^18^,^19^. Several druggable sites on ABCG2 were also reported through mutational experiments^20^,^21^. A recent docking model reported by Klepsch and collaborators was able to predict ABCB1 inhibitor with accuracy of 76%^22^. Therefore, there is a potential of applying computational models to prescreen potential inhibitors among large inhibitors library. Though the crystal structure of human ABCB1 and ABCG2 is still lacking, with the help of the two previously established homology models available to us, we performed a structure-based prescreening of 2571 inhibitors from Selleck Chemicals on ABCB1 and ABCG2. Eight ‘hits’ from virtual screening were evaluated *in vitro*. From the presumptive screening results, bafetinib (INNO-406) was identified as a potential inhibitor simultaneously on both ABCB1 and ABCG2.

Bafetinib (INNO-406) is a novel tyrosine kinase inhibitor designed based on chemical structure of imatinib for treating Bcr-Abl+ leukemia^23^,^24^. It is a potent dual function Bcr-Abl kinase and Src family kinase Lyn inhibitor^23^. Previously it is reported that bafetinib is a substrate of ABCB1 by stating bafetinib is less effective in ABCB1-overexpressing K562/D1-9 cells^25^. However, there is no published data indicating the effect of bafetinib on solid tumor cell lines that do not have Bcr-Abl. Herein in this study, through our *in silico* screening, we reported for the first time that bafetinib would be used to augment the effect of chemotherapeutical agents in ABCB1- and ABCG2-overexpressed tumor cells. The purpose of this study was to demonstrate the MDR reversal effects of bafetinib and elucidate its potential mechanism.

## Results

### Bafetinib as a potential ABCB1 and ABCG2 inhibitor through virtual screening

**Figure 1:**
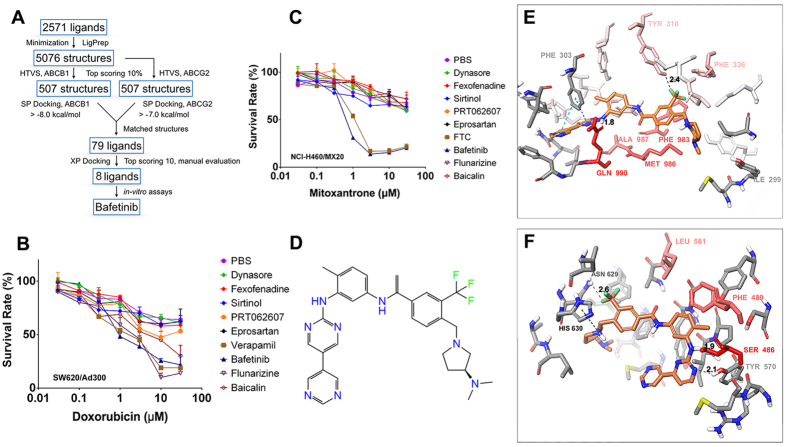
**(A)**Schematic view of virtual screening workflow on human ABCB1 and human ABCG2. **(B)** The sensitization effects of virtual screened compounds on SW620/Ad300 towards doxorubicin. **(C)** The sensitization effects of virtual screened compounds on NCI-H460/MX20 cells towards mitoxantrone. **(D)** Chemical structure of bafetinib. **(E)** Binding geometry of bafetinib into the ABCB1 binding pocket by the Glide docking algorithms. Residues of the binding pocket are highlighted in red according to their per-residue interaction scores. Residue labels are omitted for better view except important residues suggested by per-residue interaction or residues contributed in polar interactions. Carbon atoms of bafetinib are highlighted in orange. Values of the relevant distances are given in Å. **(F)** Binding geometry of bafetinib into ABCG2 binding pocket. Color scheme is same as that in panel (**E**).

Based on our previous knowledge that cell signaling inhibitors, such as tyrosine kinase inhibitors, could be possible ABC transporter inhibitors, we performed a virtual screening using inhibitor library of Selleck Chemicals provided by ZINC database. As shown in Fig. 1A, the 2571 ligands were prepared and docked into both human ABCB1 and ABCG2 homology models previously developed by our group. Seventy-nine matched ligands, whose SP docking results were in top range in both ABCB1 and ABCG2 model, were selected and re-docked into ABCB1 model using Glide XP method. The top 10 ligands from the XP run were dynasore, NVP-BHG712, flunarizine, fexofenadine, sirtinol, eprosartan, imatinib, baicalin, bafetinib and PRT062607. Among these, NVP-BHG712 and imatinib were excluded since their effects on ABCB1 and ABCG2 had been previously reported elsewhere. Flunarizine, baicalin and bafetinib were previously reported as ABCB1 substrates. Other inhibitors were purchased and examined for cytotoxicity. All inhibitors have IC_20_ values larger than 3 μM (Data not shown) therefore their reversal effects were tested at 3 μM by MTT assay (Fig. 1B,C). Only bafetinib (Fig. 1D) showed significant reversal effects on both ABCB1- and ABCG2-mediated MDR, and was then used for further study.

Bafetinib exhibited a Glide score of −11.8 kcal/mol on human ABCB1 model. As shown in Fig. 1E, bafetinib was predicted to bind into the large drug-binding cavity inside TMD of human ABCB1 through hydrophobic interaction with nearby residues. Also, two π-π stacking interactions were predicted between the two pyrimidine rings and phenyl ring of Phe 303. The NH linker formed a hydrogen bond with carbonyl group of Gln990 (-NH···OC-Gln990, 1.8 Å). The trifluoromethyl group formed a halogen bond with phenolic group of Tyr310 (-CF_3_···HO-Tyr310, 2.4 Å). Per-residue interactions simulation predicted the importance of Tyr310, Phe336, Phe983, Met986, Ala987 and Gln990 as shown in Fig. 1E.

Similarly, bafetinib was found to be stabilized into TMD of human ABCG2 mainly through hydrophobic interactions with a Glide XP score of −9.6 kcal/mol. Moreover, a cation-π interaction was found between the pyrrolidine nitrogen atom and imidazole ring of His630. The N_1_ atom of pyrimidine ring formed a hydrogen bond with phenolic group of Tyr570 (-N_1_···HO-Tyr570, 2.1 Å). The NH linker formed another hydrogen bond with hydroxyl oxygen of Ser486 (-NH···OH-Ser486, 1.9 Å). Per-residue interaction simulation predicted the importance of Ser486, Phe489 and Leu581 as shown in Fig. 1F.

### Bafetinib sensitized ABCB1-overexpressing cells to ABCB1 substrate chemotherapeutic drugs

A non-toxic concentration of bafetinib (3 μM) was selected to evaluate its reversal effects (Supplementary Information). Doxorubicin-selected SW620/Ad300 cells had a much higher IC_50_ value to doxorubicin as well as to another ABCB1-substrate paclitaxel than that in parental SW620 cells. Significant reduction in IC_50_ values were observed followed by treatment of 1 μM or 3 μM bafetinib in SW620/Ad300 cells as shown in Table 1. Meanwhile, treatment of bafetinib did not significantly change the IC_50_ values of doxorubicin and paclitaxel in parental SW620 cells. Similarly, significant decrease of IC_50_ values of doxorubicin and paclitaxel were also observed in bafetinib-treated group of transfected HEK/ABCB1 cells as compared to non-treatment group. Cisplatin, which is not a substrate of ABCB1, was used as a negative control. Verapamil at 3 μM was used as a positive control to evaluate the effects of bafetinib. Reversal effects of bafetinib at 3 μM were comparable to that of 3 μM of verapamil.

### Bafetinib sensitized both wild-type and mutant ABCG2-overexpressing cells to ABCG2 substrate chemotherapeutic drugs

It has been previously reported that mutations at Arg482 in ABCG2 might alter the substrate and inhibitor specificity of ABCG2. Therefore, we examined both wild-type (R482) and mutant (R482G and R482T) ABCG2 in the present study. As shown in Table 2, bafetinib significantly decreased the IC_50_ values of mitoxantrone and SN-38 in HEK/ABCG2-R482, HEK/ABCG2-R482G and HEK/ABCG2-R482T cells in a concentration dependent pattern. In addition, the reversal effect was also analyzed in parental NCI-H460 and ABCG2-overexpressing NCI-H460/MX20 cells. A similar reversal effect was noticed in NCI-H460/MX20 cells as shown in Table 3. Effects of bafetinib at 3 μM were found to be more potent than that of 3 μM FTC, which is used as a positive control. Decreases in IC_50_ values were also observed in NCI-H460 cells after treatment of 3 μM bafetinib and FTC.

### Bafetinib increased intracellular accumulation of doxorubicin or mitoxantrone in ABCB1- or ABCG2- overexpressing cells

**Figure 2:**
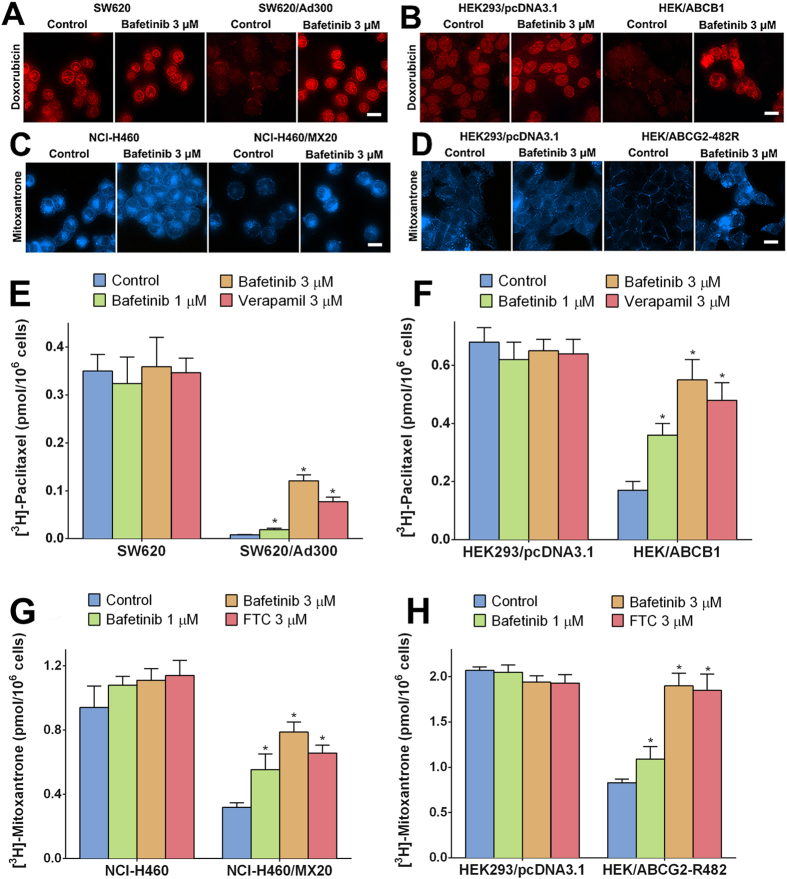
Doxorubicin accumulation fluorescence in **(A)** SW620 and SW620/Ad300 cells, **(B)** HEK293/pcDNA3.1 and HEK/ABCB1 cells pretreated or non-pretreated with bafetinib at 3 μM. Mitoxantrone accumulation fluorescence in **(C)** NCI-H460 and NCI-H460/MX20 cells, **(D**) HEK293/pcDNA3.1 and HEK/ABCG2-R482 cells pretreated or non-pretreated with bafetinib at 3 μM. Scale bar, 10 μm. The effects of bafetinib on accumulation of [^3^H]-paclitaxel in **(E)** SW620 and SW620/Ad300 cells, **(F)** HEK293/pcDNA3.1 and HEK/ABCB1 cells. Effects of bafetinib on accumulation of [^3^H]-mitoxantrone in (**G**) NCI-H460 and NCI-H460/MX20 cells, (**H**) HEK293/pcDNA3.1 and HEK/ABCG2-R482 cells. Error bars represent the SD. *p < 0.05 versus the control group. Verapamil 3 μM or FTC 3 μM is used as positive control for ABCB1- or ABCG2-overexpressing cells respectively.

In order to investigate the potential mechanism by which bafetinib sensitized ABCB1- or ABCG2-overexpressing cells, we determined the intracellular accumulation level of doxorubicin or mitoxantrone based on their own fluorescence. As shown in Fig. 2A,B, doxorubicin-associated fluorescence occurred mainly in the nuclei of SW620 and HEK293/pcDNA3.1 cells. The fluorescences of doxorubicin in resistant SW620/Ad300 and HEK/ABCB1 cells were weaker than that in parental cells while no significant nuclei accumulation was observed, several fluorescence spots were observed on the cell membranes. Pretreatment of the resistant cells with bafetinib increased the fluorescence intensity in resistant cells. The intracellular localization of doxorubicin in bafetinib-pretreated resistant cells was the same as that in the parental cells. Bafetinib had no significant effects on the intracellular accumulation or distribution of doxorubicin in the parental cells.

Similarly as shown in Fig. 2C,D, the mitoxantrone-associated fluorescence was distributed in the cytoplasm and the nuclei of parental NCI-H460 and HEK293/pcDNA3.1 cells. In contrast, fluorescence signals showed that low level of mitoxantrone was accumulated in resistant NCI-H460/MX20 and HEK/ABCG2-R482 cells. Furthermore, a membrane distribution pattern was shown in HEK/ABCG2-R482 cells. Pretreatment of the resistance cells with bafetinib also significantly increased the fluorescence intensity in resistant cells, but not significant change the intracellular accumulation pattern in the parental cells.

### Effects of bafetinib on the cellular accumulation of [^3^H]-paclitaxel or [^3^H]-mitoxantrone

[^3^H]-paclitaxel was used to further access and compare the quantitative accumulation of paclitaxel in parental and ABCB1-mediated MDR cells with or without inhibitor. As shown in Fig. 2E, intracellular [^3^H]-paclitaxel level in SW620/Ad300 cells was approximately 50-fold lower than that in the SW620 cells after 2 h incubation. Pretreatment of 1 μM bafetinib increased the intracellular [^3^H]-paclitaxel by 2-fold; while pretreatment of 3 μM bafetinib increased the intracellular [^3^H]-paclitaxel by 15.5-fold. Verapamil at 3 μM increased the intracellular [^3^H]-paclitaxel by 9.9-fold and was used as positive control. Similar trend was observed in HEK293/pcDNA3.1 and HEK/ABCB1 pair of cells as shown in Fig. 2F.

Similarly, after 2 h of accumulation, intracellular [^3^H]-mitoxantrone in NCI-H460/MX20 cells was at around 33.9% of that accumulated in NCI-H460 cells. Pretreatment of bafetinib increased the intracellular level of [^3^H]-mitoxantrone in a concentration-dependent pattern; pretreatment of 3 μM bafetinib increased the intracellular level of [^3^H]-mitoxantrone in MDR cells to 83.7% of that in parental cells. FTC at 3 μM was used as positive control inhibitor for ABCG2. Consistent pattern was observed in transfected HEK/ABCG2-R482 and its parental HEK293/pcDNA3.1 cells. Bafetinib and FTC also increased the accumulation level of [^3^H]-mitoxantrone in NCI-H460 cells but not in HEK293/pcDNA3.1 cells.

### Effects of bafetinib on the efflux time-course of [^3^H]-paclitaxel or [^3^H]-mitoxantrone

**Figure 3:**
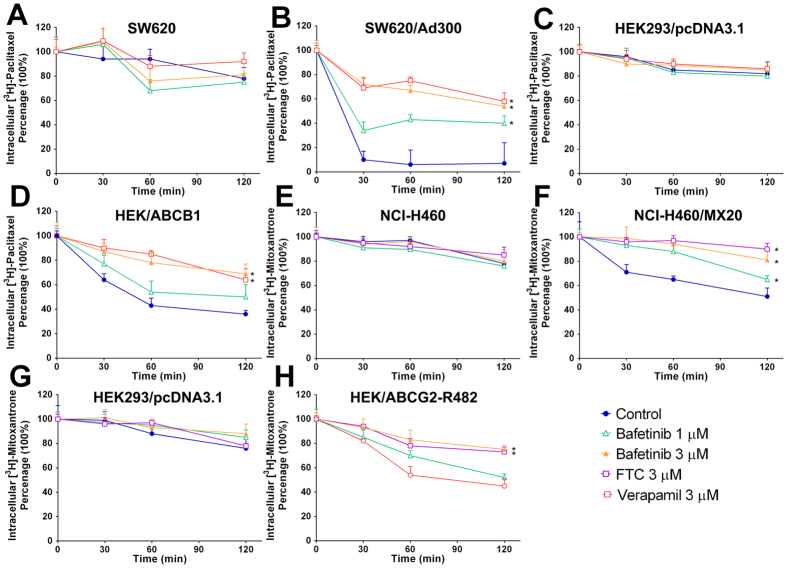
Time courses verses percentage of intracellular [^3^H]-paclitaxel remaining was plotted to show effects of bafetinib in (**A**) SW620, **(B)** SW620/Ad300, **(C)** HEK293/pcDNA3.1 and **(D**) HEK/ABCB1 cells. Time courses verses percentage of intracellular [^3^H]-mitoxantrone remaining was plotted in treated and non-treated **(E)** NCI-H460, **(F)** NCI-H460/MX20, **(G)** HEK293/pcDNA3.1 and **(H)** HEK/ABCG2-R482 cells. *p < 0.05 versus the control group. Verapamil 3 μM or FTC 3 μM is used as positive control for ABCB1- or ABCG2-overexpressing cells respectively.

[^3^H]-paclitaxel efflux occurred in the MDR and parental cells after removal of [^3^H]-paclitaxel from the culture medium. As shown in Fig. 3A, SW620 cells lost 22% of the normalized intracellular [^3^H]-paclitaxel at the end of 2 h efflux. Meanwhile, more than 90% loss of normalized [^3^H]-paclitaxel accumulation was observed in SW620/Ad300 cells (Fig. 3B). Consistently, only less than a 20% loss of normalized [^3^H]-paclitaxel occurred in HEK293/pcDNA3.1 cells (Fig. 3C). In contrast, HEK/ABCB1 cells lost more than 60% of accumulated [^3^H]-paclitaxel (Fig. 3D). Treatment with bafetinib or ABCB1 inhibitor verapamil increased the retention of [^3^H]-paclitaxel in ABCB1-overexpressing cells. Effects of bafetinib followed a concentration-dependent pattern and 3 μM bafetinib exhibited 54% or 62% of [^3^H]-paclitaxel retention in SW620/Ad300 cells or HEK/ABCB1 cells, respectively. Neither bafetinib nor verapamil significantly altered efflux pattern in parental cells.

Furthermore, as shown in Fig. 3E,F, untreated NCI-H460/MX20 cells lost 49% of the normalized intracellular [^3^H]-mitoxantrone, while its parental NCI-H460 lost 22% of the normalized [^3^H]-mitoxantrone. With the treatment of 3 μM bafetinib, only 19% of intracellular accumulated [^3^H]-mitoxantrone was lost after same time of efflux as compared to non-treated group. Consistently as shown in Fig. 3G,H, treatment with 3 μM bafetinib significantly increased the [^3^H]-mitoxantrone retention in HEK/ABCG2-R482 cells however not in HEK293/pcDNA3.1 cells. FTC at 3 μM was used to evaluate inhibitory effects of bafetinib on ABCG2 as a positive control.

### Bafetinib did not change the expression level and the intracellular localization of ABCB1 or ABCG2 in cells overexpressing ABCB1 or ABCG2 respectively

**Figure 4:**
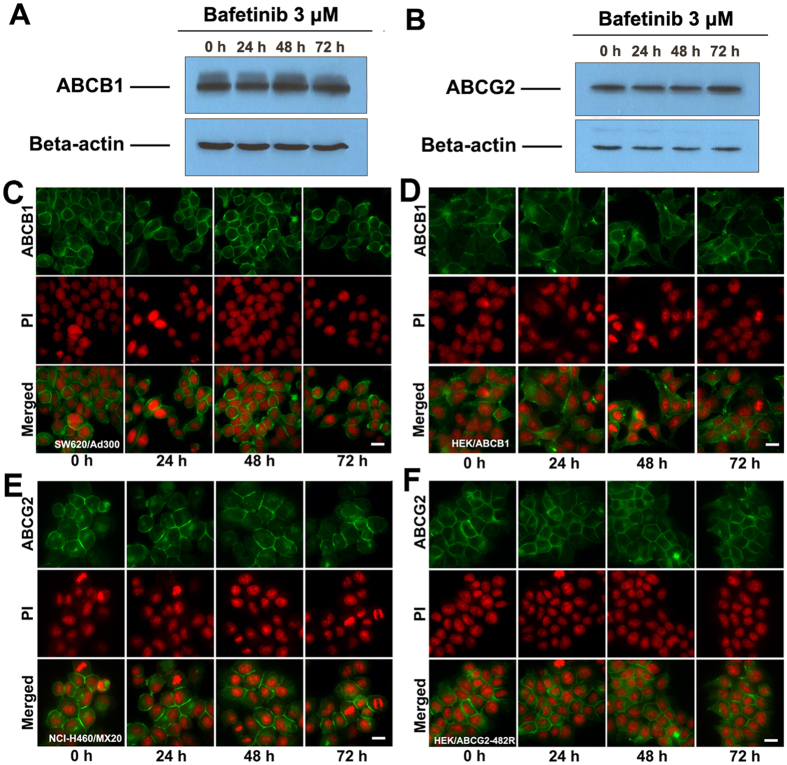
**(A)**The effect of bafetinib at 3 μM on the expression levels of ABCB1 in SW620/Ad300 cells for 24, 48 and 72 h. **(B)** The effect of bafetinib at 3 μM on the expression level of ABCG2 in NCI-H460/MX20 cells for 24, 48 and 72 h. Equal amounts of total cell lysate were used for each sample. The effect of bafetinib at 3 μM on the subcellular localization of ABCB1 in ABCB1-overexpressing **(C)** SW620/Ad300 cells and **(D)** HEK/ABCB1 cells for 24, 48 and 72 h. The effect of bafetinib at 3 μM on the subcellular localization of ABCG2 in ABCG2-overexpressing **(E)** NCI-H460/MX20 cells and **(F)** HEK/ABCG2-R482 cells. Scale bar, 10 μm. PI (propidium Iodide, red) counterstains the nuclei.

ABCB1 and ABCG2 expression levels were demonstrated with Western blotting analysis. As previously reported, the monoclonal antibody C219 specifically detects a 170-kDa ABCB1 in SW620/Ad300 cells and the monoclonal antibody BXP-21 detects a 72-kDa ABCG2 in NCI-H460/MX20 cells^26^. Samples were taken at a 24 h interval after exposure to 3 μM bafetinib. As shown in Fig. 4A,B, the treatment of 3 μM bafetinib up to 72 hours did not significantly change the expression level of ABCB1 or ABCG2 in SW620/Ad300 or NCI-H460/MX20 cells respectively at any time points. Beta-actin at 42 kDa was used as loading control for Western blotting analysis.

As shown in Fig. 4C,D by immunofluorescence staining, ABCB1 transporters showed a membrane-located pattern in SW620/Ad300 and HEK/ABCB1 cells. The incubation of these cells with 3 μM bafetinib did not significantly alter the subcellular distribution pattern of ABCB1 when compared to control at any time point at 24 h interval. ABCG2 transporters showed a similar membrane-located pattern in NCI-H460/MX20 cells (Fig. 4E) and HEK/ABCG2-R482 cells (Fig. 4F), while few transporters are located in the cytoplasm near nuclei. These distribution patterns are not altered by treatment of 3 μM bafetinib.

### Bafetinib stimulated ATPase activity of ABCB1 and inhibited ATPase activity of ABCG2

**Figure 5:**
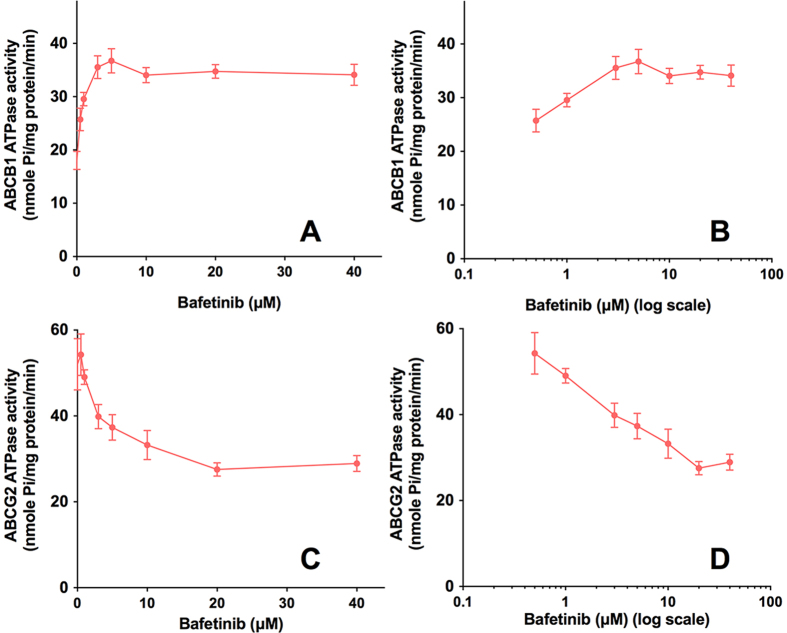
Effect of bafetinib on orthovanadate (Vi)-sensitive ABCB1 ATPase activity with increased concentration of bafetinib (0–40 μM). Concentration of bafetinib was plotted at **(A**) linear or **(B)** log scale. Effect of bafetinib on Vi-sensitive ABCG2 ATPase activity with increased concentration of bafetinib (0–40 μM). Concentration of bafetinib was plotted at **(C)** linear or **(D)** log scale.

ABCB1- or ABCG2-mediated ATP hydrolysis in the presence of bafetinib at various concentrations from 0 to 40 μM was measured to assess the effect of bafetinib on the ATPase activity of ABCB1 or ABCG2, respectively. Bafetinib stimulated the ATPase activity of ABCB1 in a concentration-dependent manner, with a maximal stimulation of 2-fold of the basal activity as shown in Fig. 5A,B. Interestingly, bafetinib inhibited the ATPase activity of ABCG2 in a concentration-dependent manner, with a maximal inhibition of 47% of the basal activity (Fig. 5C,D). Figure 5 demonstrates that the concentration of bafetinib required to obtain 50% of maximal stimulation or inhibition is 0.64 μM or 2.97 μM for ABCB1 or ABCG2 respectively.

## Discussion

As previously reported, several small molecule cell signaling inhibitors, particularly tyrosine kinase inhibitors, are potent inhibitors of ABC transporters^3^,^11^,^27^. Therefore, it is of great value and interest to us to discover novel inhibitors of ABC transporter from these kinase inhibitors. As cancer cells may develop MDR via different types of ABC transporters, inhibitor for single ABC transporter may not be effective. For example colon cancer cell may become MDR via ABCB1 (SW620/Ad300 cells) or via ABCG2 (S1-M1-80 cells). Therefore, in this study, we aimed to find the ‘hits’ for both ABCB1 and ABCG2. With the 3D structures of human homology ABCB1 and ABCG2, we were able to perform a structure-based *in silico* screening to filter possible inhibitors through large ligand database. Our top scoring ligands screened through our virtual screening protocol included previously reported potent ABCB1 and ABCG2 inhibitors imatinib^28^ and a weak inhibitor NVP-BHG712^29^. flunarizine^30^, and ABCB1 substrate baicalin^31^. These results indicated great potential and validity of our screening protocol. Among the candidates, bafetinib at its non-toxic concentration of 3 μM was demonstrated as a potent inhibitor for both ABCB1 and ABCG2.

Our MTT assays showed that doxorubicin-selected SW620/Ad300 cells also acquired resistance towards paclitaxel. This MDR cells had shown to be sensitized toward doxorubicin or paclitaxel by pre-incubation of bafetinib in a concentration-dependent manner. Beside the fact that bafetinib is reported as a potential ABCB1 substrate, the effects of bafetinib were clearly related to the ABCB1 overexpressed in SW620/Ad300 through our study for the following reasons. First, bafetinib, at its non-toxic concentration, did not alter the IC_50_ values of doxorubicin or paclitaxel on parental SW620 cells, in which ABCB1 is not expressed^32^,^33^. Second, bafetinib at its non-toxic concentration did not sensitize SW620/Ad300 cells towards cisplatin, which is not a substrate of ABCB1. Last, bafetinib exhibited similar reversal effects on ABCB1-transfected HEK/ABCB1 cells, in which ABCB1 is the sole contributor for multidrug resistance. Therefore, bafetinib reversed MDR in SW620/Ad300 and HEK/ABCB1 cells by targeting at ABCB1 transporters.

Likewise, effect of bafetinib on ABCG2-overexpressing MDR cells NCI-H460/MX20 was related to the ABCG2 transporter. The decreases of IC_50_ values in parental NCI-H460 cells may due to moderate level expression of ABCG2 as previously reported^26^,^34^. Moreover, site-mutation on target might produce significant differences in the effectiveness of inhibitor, such as T315I mutation will inactive bafetinib at Bcr-Abl^23^. Mutation at position 482 in ABCG2 has been previously demonstrated for affecting inhibitor efficacy such as novobiocin^35^. Our results showed that bafetinib could inhibit the activity of both wild-type (R482) and two mutant variant (R482G and R482T) of ABCG2. Also, our docking simulation of bafetinib at ABCG2 did not predict great contribution of Arg 482 in bafetinib binding.

Furthermore, our study indicated a trend of correlation exists between the potency of doxorubicin or mitoxantrone and the intracellular accumulation of the compounds. Consistent with previous report, doxorubicin was mainly accumulated in the cellular nuclei in parental SW620 and HEK293/pcDNA3.1 cells^36^. The low level of doxorubicin accumulated in MDR cells may account for its higher IC_50_ values. Also, light fluorescence detected doxorubicin near cellular membrane suggested that doxorubicin was pumped out of the cells. Bafetinib treatment significantly increased the intracellular doxorubicin accumulation, providing clues for the mechanisms of its reversal effects. The level of doxorubicin accumulation was further confirmed and quantified using another ABCB1 substrate [^3^H]-paclitaxel. Moreover, by plotting the efflux time-course, it is clear that the resistance in MDR cells is on account of an accelerated efflux and bafetinib, as well as verapamil, acts by inhibiting the drug efflux. Similarly, mitoxantrone distributed in both cellular nuclei and cytoplasm in parental NCI-H460 and HEK293/pcDNA3.1 cells^37^. The membrane accumulated pattern of mitoxantrone in the MDR cells, especially transfected HEK/ABCG2-R482, suggested a process of drug efflux. Treatment of bafetinib in MDR cells was able to reverse the intracellular mitoxantrone accumulation to the level which is similar within the parental cells. Therefore, we concluded that bafetinib reversed the ABCB1- or ABCG2-mediated MDR by inhibiting the transported mediated drug efflux, thereby increasing the intracellular drug accumulation.

Connections between ABC transporters and other cell signaling transducer proteins, such as PKA and PKC, have been previously reported^38^. As membrane-bound transporter, ABCB1 or ABCG2 might lose their function by being down-regulated or translocated into the cytoplasm upon treatment, which could have potentiated the reversal effects of bafetinib. These hypotheses were examined by our Western blotting and immunofluorescence studies. No change in protein expression level or in subcellular location was observed when ABCB1- or ABCG2-overexpressing cells were treated with bafetinib at 3 μM for 24, 48 and 72 h, respectively. Overall, the reversal effects of bafetinib on ABCB1 or ABCG2 is therefore related to its direct inhibition of function of these ABC transporters. It was hypothesized that bafetinib could interact with the ATP-binding pocket in NBD of ABC transporters, similar to its binding to the ATP-binding pocket on the BCR-Abl kinase. Although inhibitory ATPase activity on ABCG2 as increasing concentration of bafetinib was observed, our docking simulation predicted low score of bafetinib at ABCG2 ATP binding site (−5.660 kcal/mol)^39^. On the other hand, increased ATPase activity on ABCB1 upon increasing concentration of bafetinib suggested that bafetinib might competitively inhibit ABCB1 by binding to the drug-binding pocket in TMD. Since NBDs of ABCB1 and ABCG2 exhibited high levels of sequence homology and possessed the similar conserved secondary structure topology as previous reported^40^, it is believed that bafetinib bound and stabilized with TMD of ABCG2 instead of being transported. Therefore, bafetinib may serve as an actual inhibitor of ABCG2 while as an competitive substrate of ABCB1. The difference in TMDs of ABCB1 and ABCG2 accounts for their difference in substrates recognition as well as their responses to bafetinib binding.

In conclusion, this study report that bafetinib reverse ABCB1- and ABCG2-mediated MDR by directly inhibiting their efflux functions. These results tentatively suggest that bafetinib could be used in patients with MDR to augment the clinical response to chemotherapeutic agents that are substrates of ABCB1 and ABCG2.

## Materials and Methods

### Reagents

Bafetinib, dynasore, fexofenadine, sirtinol, eprosartan mesylate and PRT062607 were purchased or provided as samples from Selleck Chemicals (Houston, USA). Dulbecco’s modified Eagle’s Medium (DMEM), fetal bovine serum (FBS), penicillin/streptomycin and trypsin 0.25% were purchased from Hyclone (GE Healthcare Life Science, Pittsburgh, PA). Bovine Serum Albumin (BSA), monoclonal antibody C219 (against ABCB1), monoclonal antibody BA3R (against beta-actin), Alexa Fluor 488 conjugated goat anti-mouse IgG secondary antibody, RNase A and SN-38 were purchased from Thermo Fisher Scientific Inc. (Rockford, IL). Monoclonal antibody BXP-21 (against BCRP) was purchased from GeneTex (Irvine, CA). HRP-labeled rabbit anti-mouse secondary IgG was purchased from Santa Cruz Biotechnology (Dallas, TX). Monoclonal antibody P7965 (against ABCB1), paclitaxel, doxorubicin, cisplatin, mitoxantrone, verapamil, 3-(4,5-dimethylthiazol-yl)-2,5-diphenyltetrazolium bromide (MTT), dimethylsulfoxide (DMSO), Triton X-100, propidium iodide and paraformaldehyde were products from Sigma-Aldrich (St. Louis, MO). [^3^H]-paclitaxel (15 Ci/mmol) and [^3^H]-mitoxantrone (2.5 Ci/mmol) were purchased from Moravek Biochemicals, Inc (Brea, CA). The chemicals used in ATPase assay not included in the kit were same as those in our previous study^33^. Fumitremorgin C (FTC) was a gift from Dr. Susan Bates (NIH, Bethsda, MD).

### Cell lines and cell culture

Human colon cancer cell line SW620 and its doxorubicin-selected ABCB1-overexpressing SW620/Ad300 cell line were used for ABCB1 reversal study, non-small cell lung cancer cell line NCI-H460 and its mitoxantrone-selected ABCG2-overexpressing NCI-H460/MX20 cells were used in ABCG2 reversal study. These cells were kindly provided by Drs. Susan Bates and Robert Robey (NIH, Bethesda, MD). HEK293/pcDNA3.1, HEK/ABCB1, HEK/ABCG2-482R, HEK/ABCG2-R482G and HEK/ABCG2-R482T were established by transfecting HEK293 cells with either empty pcDNA3.1 vector (HEK293/pcDNA3.1) or vector containing full length *ABCB1* (HEK/ABCB1), wild type *ABCG2* (HEK/ABCG2-R482) or mutant *ABCG2* (HEK/ABCG2-R482G and HEK/ABCG2-R482T), and were cultured in a medium containing 2 mg/mL of G418. ABCB1-transfected cell lines are provided by Dr. Suresh V. Ambudkar (NIH, Bethesda, MD) and ABCG2-transfected cell lines are provided by Drs. Susan Bates and Robert Robey (NIH, Bethesda, MD). All the cell lines were grown as adherent monolayers in flasks with DMEM supplemented with 10% FBS and 1% penicillin/streptomycin in a humidified incubator containing of 5% CO_2_ at 37 °C. All cells were tested every 12 weeks for detection of mycoplasma contamination.

### Virtual screening and molecular docking

Selleck chemicals ligand 2D structures database was downloaded from ZINC^41^ (San Francisco, CA) and were prepared using our previous molecular modeling protocols^33^. The human ABCB1 homology model based on refined mouse ABCB1 (RCSB ID: 4M1M) was kindly provided by S. Aller and the grid of 25 Å was refined as previously described^19^. Our previous grid on human homology ABCG2 on centroid of Arg482 was used for docking on ABCG2^42^. Glide HTVS (high throughput virtual screening) docking protocol was followed to dock all prepared 5076 structures into ABCB1 and ABCG2 (Schrödinger, LLC, New York, NY). Top scoring (Glide score) 10% ligands of ABCB1 or ABCG2 were selected respectively and were subjected to Glide SP (standard precision) docking. Ligands with Glide SP score more than −8.0 kcal/mol or −7.0 kcal/mol were kept respectively for ABCB1 and ABCG2. 79 ligands were selected as overlaps between ABCB1 and ABCG2 SP docking results and then submitted to Glide XP (extra precision) docking (Schrödinger, LLC, New York, NY). The top 10 results were examined visually and 8 compounds were acquired for reversal MTT assays. All computations were carried out on a 6-core Intel Xeon Processor with a Mac OS. A schematic view of virtual screening processes was shown in Fig. 1A.

### Cytotoxicity by MTT assay

Modified MTT colorimetric assay was used to detect the cytotoxicity of anticancer drugs with or without modulator agents. 5 × 10^3^ cells were seeded evenly into each well in 96-well microplates and cultured overnight. Bafetinib and parallel control modulators were added 1 h prior. Chemotherapeutic drugs were then added into the designated wells by concentration gradient. After 72 h of incubation, 20 μL of MTT solution (4 mg/mL) was added into each well, then the microplate was further incubated for 4 h. Subsequently, the medium was aspirated and 100 μL of DMSO was added to dissolve the formazan crystal in each well. The absorbance was determined at 570 nm by the OPSYS microplate reader (Dynex Technology, Chantilly, VA). The IC_50_ values were calculated from the survival curves using modified Bliss method. Verapamil was used as a positive control for ABCB1 overexpressing cell lines. FTC was used as a positive control for ABCG2 overexpressing cell lines.

### Doxorubicin or mitoxantrone intracellular accumulation and effects of bafetinib

Cells were seeded on sterile coated coverslips and were allowed to grow overnight. On the following day, cells were washed with phosphate-buffered saline (PBS), incubated with or without 3 μM of Bafetinib for 1 h before being treated with 5 μM doxorubicin or mitoxantrone for 2 h, and then examined using fluorescence microscopy. SW620, HEK293/pcDNA3.1, SW620/Ad300, and HEK/ABCB1 cells received doxorubicin treatment. NCI-H460, HEK293/pcDNA3.1, NCI-H460/MX20 and HEK/ABCG2-R482 were tested for mitoxantrone accumulation.

### [^3^H]-paclitaxel or [^3^H]-mitoxantrone accumulation assay

The accumulation of [^3^H]-paclitaxel in SW620, SW620/Ad300, HEK293/pcDNA3.1 and HEK/ABCB1 was measured in the presence or absence of inhibitors. Cells were trypsinized, resuspended and pre-incubated with either PBS, bafetinib (1 μM and 3 μM) or verapamil (3 μM) for 1 h. Subsequently, cells were incubated in the medium containing 0.1 μM [^3^H]-paclitaxel for 2 h in the presence of above treatment. After washing three times with ice cold PBS, the cells were lysised and placed in 5 mL scintillation fluid. Radioactivity was measured in the Packard TRI-CARB 1900CA liquid scintillation analyzer (Packard Instrument, Downers Grove, IL). Same protocol was followed by using 0.01 μM [^3^H]-mitoxantrone in NCI-H460, NCI-H460/MX20, HEK293/pcDNA3.1 and HEK293/ABCG2-R482 cells.

### [^3^H]-paclitaxel or [^3^H]-mitoxantrone efflux assay

To measure the [^3^H]-paclitaxel drug efflux, cells were incubated sequentially with medium with 0.1 μM [^3^H]-paclitaxel for 2 h (accumulation phase), and then [^3^H]-paclitaxel-free medium for 2 h (efflux phase). Serial aliquots at 0, 30, 60 and 120 min in efflux phase were taken and analyzed as previously described^33^. To study the effect of bafetinib on the time course of [^3^H]-paclitaxel efflux, cells were pre-exposed to 1 μM or 3 μM bafetinib for 1 hour before being treated with [^3^H]-paclitaxel containing medium. Then bafetinib and paralled control inhibitor were also added to each phase, respectively. Same protocol was followed by using 0.01 μM [^3^H]-mitoxantrone to study ABCG2-mediated drug efflux.

### Preparation of total cell lysates and Western blotting

Cell extracts were prepared by incubating on ice with lysis buffer (10 mM Tris, 1 mM EDTA, 0.1% SDS, 150 mM NaCl, 1% Triton-X and protease inhibitor cocktail) for 20 min, followed by centrifugation at 12,000 × g at 4 °C for 5 min. The supernatant containing total cell lysate was collected and protein concentration was determined by bicinchoninic acid (BCA^TM^) based protein assay (Thermo Scientific, Rockford, IL). Equal amount of total cell lysates were loaded and separated by SDS-polyacrylamide gel electrophoresis and transferred to a polyvinylidene difluoride membrane. For ABCB1-overexpressing cells, the blot was then probed with the primary antibody C219 (dilution 1:200) and primary antibody BA3R (dilution 1:500). For ABCG2-overexpressing cells, the blot was probed with primary antibody BXP-21 (dilution 1:500) and primary antibody BA3R (dilution 1:500). Membranes were then further incubated with HRP-conjugated secondary antibody. The signal was detected using enhanced chemiluminescence and exposure of X-ray film.

### ABCB1 and ABCG2 ATPase assays

The ABCB1- and ABCG2-associated ATPase activities were measured by PREDEASY ATPase Kits with modified protocols. Basically, the ABCB1- or ABCG2-membranes were thawed and diluted. Sodium orthovanadate (Na_3_VO_4_) was used as an ATPase inhibitor. Various concentrations of bafetinib were incubated with membranes for 5 min, then the ATPase reactions were initiated by adding 5 mM Mg^2+^ ATP. After incubation at 37 for 40 min with brief mixing, luminescence signals of Pi were initiated and measured. The changes of relative light units were determined by comparing Na_3_VO_4_-treated samples with bafetinib-treated groups.

### Immunofluorescence of ABCB1 or ABCG2

Cells were seeded on sterile coated coverslips and were allowed to grow overnight, followed by treatment with 3 μM bafetinib for 24, 48 and 72 h respectively. The cells were then washed with ice-cold PBS, fixed in 4% paraformaldehyde, permeatabilized by 0.1% Triton X-100 and then blocked by 6% BSA for 1 h. Subsequently, cells were incubated with either monoclonal antibody P7965 against ABCB1 (1:400, for SW620/Ad300 and HEK/ABCB1 cells) or monoclonal antibody BXP-21 against ABCG2 (1:400, for NCI-H460/MX20 and HEK/ABCG2-R482) overnight, followed by Alexa Fluor 488 conjugated secondary antibody (1:2000) for 1 h. Propidium iodide/RNase solution was applied to counterstain the nuclei. Cells were mounted onto glass slides and then examined under fluorescence microscopy.

### Fluorescence microscopy

Images were collected using a Nikon TE-2000S fluorescence microscope with an APO 60× oil immersion lens (Nikon). Propidium iodide, mitoxantrone and doxorubicin fluorescence were excited and collected with Nikon TRITC HYQ excitation filter combination. The fluorescence of mitoxantrone was represented using pseudo color (blue) to represent the physical blue color of mitoxantrone liquid. Alexa Fluor 488 fluorescence was excited and collected with Nikon B1-E filter combinations. The same fluorescence settings (excitation, lamp power, detector gain and exposure time) were used to image different samples.

### Statistics

All data are expressed as mean ± S.D. from three or more experiments and statistically evaluated by one-way ANOVA. Differences were considered significant when *p* < 0.05.

## Additional Information

**How to cite this article**: Zhang, Y.-K. *et al*. Bafetinib (INNO-406) reverses multidrug resistance by inhibiting the efflux function of ABCB1 and ABCG2 transporters. *Sci. Rep.* **6**, 25694; doi: 10.1038/srep25694 (2016).

## Supplementary Material

Supplementary Information

## Figures and Tables

**Table 1:** Bafetinib reverses the ABCB1-mediated drug resistance to doxorubicin and paclitaxel in ABCB1-overexpressing cell lines.

Treatment	IC_50_ value ± SD^a^			
	SW620 (μM, Resistance Fold^b^)	SW620/Ad300 (μM, Resistance Fold^b^)	HEK293/pcDNA3.1 (nM, Resistance Fold^b^)	HEK/ABCB1 (nM, Resistance Fold^b^)
Doxorubicin	0.11 ± 0.02 (1.0)	46.2 ± 2.7 (421.3)	67.9 ± 4.1 (1.0)	814.6 ± 122.1 (12.0)
+ Bafetinib 1 μM	0.11 ± 0.03 (1.0)	11.3 ± 1.9 (103.6)*	53.1 ± 9.3 (0.8)	537.6 ± 119.3 (7.9)*
+ Bafetinib 3 μM	0.08 ± 0.02 (0.7)	1.7 ± 0.3 (15.4)*	78.7 ± 6.3 (1.2)	81.4 ± 35.2 (1.2)*
+ Verapamil 3 μM	0.09 ± 0.02 (0.8)	2.3 ± 0.2 (21.0)*	72.1 ± 7.0 (1.1)	137.4 ± 27.1 (2.0)*
				
Paclitaxel	0.07 ± 0.01 (1.0)	14.6 ± 2.3 (208.6)	33.8 ± 2.7 (1.0)	1352.2 ± 42.2 (40.0)
+ Bafetinib 1 μM	0.08 ± 0.01 (1.1)	5.5 ± 0.8 (78.6)*	37.9 ± 3.5 (1.1)	412.2 ± 27.9 (12.2)*
+ Bafetinib 3 μM	0.08 ± 0.02 (1.1)	0.3 ± 0.06 (4.3)*	44.7 ± 3.3 (1.3)	58.0 ± 2.6 (1.7)*
+ Verapamil 3 μM	0.09 ± 0.02 (1.3)	0.8 ± 0.1 (11.4)*	35.2 ± 2.3 (1.0)	78.6 ± 11.8 (2.3)*
				
Cisplatin	1.8 ± 0.3 (1.0)	1.7 ± 0.2 (0.9)	817.9 ± 96.2 (1.0)	1153.5 ± 170.6 (1.4)
+ Bafetinib 1 μM	1.3 ± 0.1 (0.7)	1.8 ± 0.1 (1.0)	865.3 ± 73.7 (1.1)	971.3 ± 72.5 (1.2)
+ Bafetinib 3 μM	1.5 ± 0.1 (0.8)	1.7 ± 0.2 (0.9)	840.1 ± 68.2 (1.0)	933.3 ± 66.4 (1.1)
+ Verapamil 3 μM	1.7 ± 0.2 (0.9)	2.0 ± 0.5 (1.1)	822.5 ± 69.6 (1.0)	936.7 ± 51.3 (1.1)

^a^IC_50_ values are represented as mean ± SD of three independent experiments performed in triplicate. ^b^Resistance fold was calculated by dividing the IC_50_ values of substrates in the presence or absence of inhibitor by the IC_50_ of parental cells without inhibitor.

^*^p < 0.05 versus no inhibitor group.

**Table 2:** Bafetinib reverse the ABCG2-mediated drug resistance to mitoxantrone and SN-38 in wild-type and mutant *ABCG2*-transfected cell lines.

Treatment	IC_50_ value ± SD^a^ (nM, Resistance Fold^b^)			
	HEK293/pcDNA3.1	HEK/ABCG2-R482	HEK/ABCG2-R482G	HEK/ABCG2-R482T
Mitoxantrone	59.1 ± 7.2 (1.0)	310.1 ± 23.7 (5.2)	677.6 ± 70.8 (11.5)	470.7 ± 21.6 (7.9)
+ Bafetinib 1 μM	53.7 ± 5.5 (0.9)	122.0 ± 14.9 (2.1)*	186.9 ± 31.8 (3.2)*	105.2 ± 12.7 (1.8)*
+ Bafetinib 3 μM	57.4 ± 5.2 (0.9)	82.5 ± 4.9 (1.4)*	65.8 ± 3.4 (1.1)*	52.6 ± 6.8 (0.9)*
+ FTC 3 μM	76.2 ± 9.1 (1.3)	94.2 ± 7.8 (1.6)*	73.8 ± 7.3 (1.3)*	64.1 ± 4.9 (1.1)*
				
SN-38	2.2 ± 0.3 (1.0)	25.1 ± 2.4 (11.4)	21.7 ± 3.6 (9.9)	25.3 ± 4.5 (11.5)
+ Bafetinib 1 μM	2.8 ± 0.5 (1.3)	14.9 ± 0.9 (6.8)*	11.5 ± 0.9 (5.2)*	12.7 ± 2.3 (5.8)*
+ Bafetinib 3 μM	2.5 ± 0.1 (1.1)	2.8 ± 0.3 (1.3)*	2.7 ± 0.2 (1.2)*	3.6 ± 0.5 (1.6)*
+ FTC 3 μM	2.1 ± 0.2 (1.0)	2.2 ± 0.3 (1.0)*	2.4 ± 0.1 (1.1)*	2.3 ± 0.3 (1.0)*
				
Cisplatin	864.3 ± 77.2 (1.0)	849.5 ± 97.8 (1.0)	889.9 ± 117.5 (1.0)	887.3 ± 78.3 (1.0)
+ Bafetinib 1 μM	885.5 ± 123.6 (1.0)	857.6 ± 107.7 (1.0)	845.5 ± 75.3 (1.0)	849.5 ± 68.1 (1.0)
+ Bafetinib 3 μM	959.6 ± 133.7 (1.1)	933.0 ± 119.4 (1.1)	877.6 ± 83.9 (1.0)	925.5 ± 102.9 (1.1)
+ FTC 3 μM	808.5 ± 88.7 (0.9)	846.8 ± 87.8 (1.0)	815.2 ± 82.5 (0.9)	801.1 ± 112.1 (0.9)

^a^IC_50_ values are represented as mean ± SD of three independent experiments performed in triplicate. ^b^: Resistance fold was calculated by dividing the IC_50_ values of substrates in the presence or absence of inhibitor by the IC_50_ of parental cells without inhibitor.

^*^p < 0.05 versus no inhibitor group.

**Table 3:** Bafetinib reverses the ABCG2-mediated drug resistance to mitoxantrone and SN-38 in parental and drug-selected ABCG2-overexpressing cell lines.

Treatment	IC_50_ value ± SD^a^ (μM, Resistance Fold^b^)	
	NCI-H460	NCI-H460/MX20
Mitoxantrone	0.06 ± 0.01 (1.0)	35.4 ± 5.4 (590.1)
+ Bafetinib 1 μM	0.05 ± 0.01 (1.3)	1.6 ± 0.5 (26.7)*
+ Bafetinib 3 μM	0.04 ± 0.003 (0.7)*	0.7 ± 0.1 (11.7)*
+ FTC 3 μM	0.04 ± 0.007 (0.7)*	1.4 ± 0.2 (23.3)*
		
SN-38	0.07 ± 0.01 (1.0)	8.7 ± 1.7 (124.7)
+ Bafetinib 1 μM	0.08 ± 0.01 (1.1)	2.3 ± 0.6 (32.9)*
+ Bafetinib 3 μM	0.04 ± 0.005 (0.6)*	0.4 ± 0.1 (5.7)*
+ FTC 3 μM	0.05 ± 0.003 (0.7)*	0.9 ± 0.2 (12.9)*
		
Cisplatin	1.2 ± 0.2 (1.0)	1.1 ± 0.3 (1.0)
+ Bafetinib 1 μM	1.1 ± 0.1 (0.9)	1.1 ± 0.2 (1.0)
+ Bafetinib 3 μM	1.3 ± 0.1 (1.1)	1.3 ± 0.2 (1.2)
+ FTC 3 μM	1.1 ± 0.3 (0.9)	1.4 ± 0.3 (1.3)

^a^IC_50_ values are represented as mean ± SD of three independent experiments performed in triplicate. ^b^Resistance fold was calculated by dividing the IC_50_ values of substrates in the presence or absence of inhibitor by the IC_50_ of parental cells without inhibitor.

^*^p < 0.05 versus no inhibitor group.
