# Supplementary material for: Bafetinib (INNO-406) reverses multidrug resistance by inhibiting the efflux function of ABCB1 and ABCG2 transporters
Source: Sci Rep. 2016 May 9;6:25694. doi: 10.1038/srep25694 (PMC4860574; doi:10.1038/srep25694)
Supplement: Supplementary Information [file srep25694-s1.pdf]

Supplemental Data

Bafetinib (INNO-406) reverses multidrug resistance by inhibiting the efflux function of ABCB1 and ABCG2 transporters

Yun-Kai Zhang[1], Guan-Nan Zhang[1], Yi-Jun Wang[1], Bhargav A. Patel[1], Tanaji T. Talele[1], Dong-Hua Yang[1] and Zhe-Sheng Chen[1,*]

[1]Department of Pharmaceutical Sciences, College of Pharmacy and Health Sciences, St. John's University, Queens, NY 11439, USA

Corresponding Author:

*Dr. Zhe-Sheng Chen, M.D., Ph.D., Department of Pharmaceutical Sciences, St. John's University, Queens, New York, 11439, USA. Tel: 1-718-990-1432, Fax: 1-718-990-1877. Email: chenz@stjohns.edu

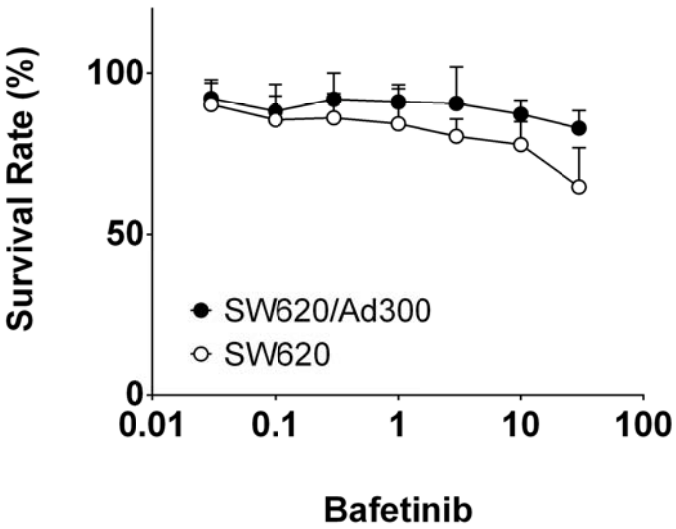

Supplemental Figure S1. Cytotoxicity of bafetinib in parental SW620 and ABCB1-overexpressing SW620/Ad300 cells. X axis in μM.

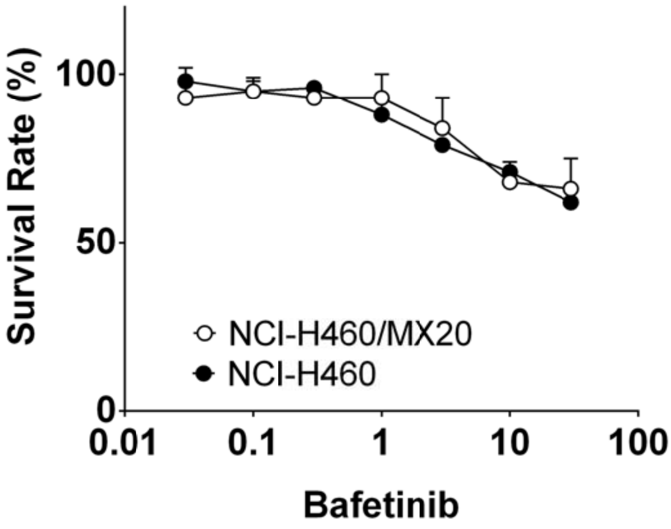

Supplemental Figure S2. Cytotoxicity of bafetinib in parental NCI-H460 and ABCG2-overexpressing NCI-H460/MX20 cells. X axis in μM.

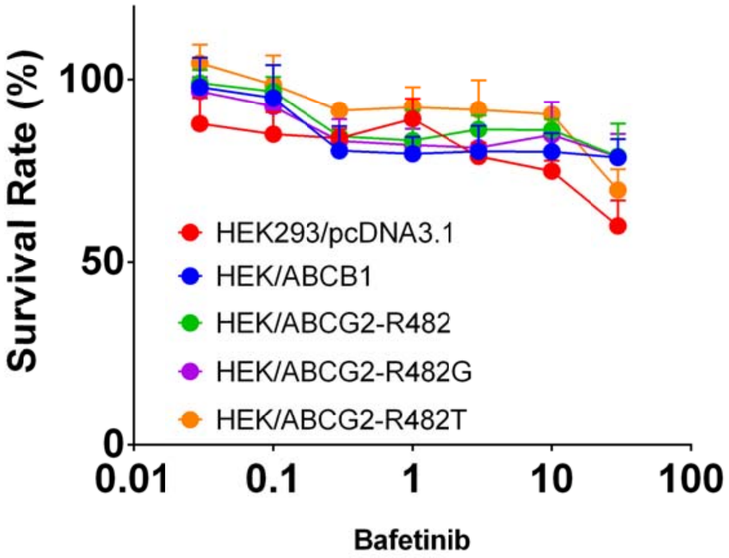

Supplemental Figure S3. Cytotoxicity of bafetinib in parental HEK293/pcDNA3.1, ABCB1-transfected and ABCG2-transfected cell lines. X axis in μM.

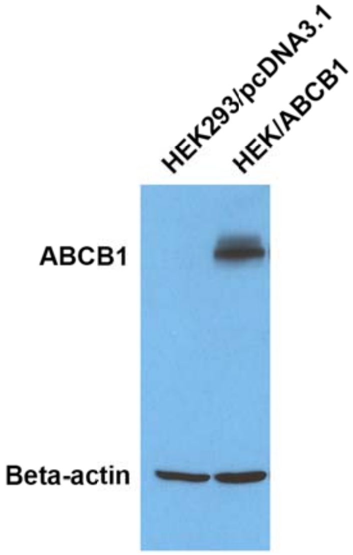

Supplemental Figure S4. Expression of ABCB1 in HEK293/pcDNA3.1 and HEK/ABCB1 cells.

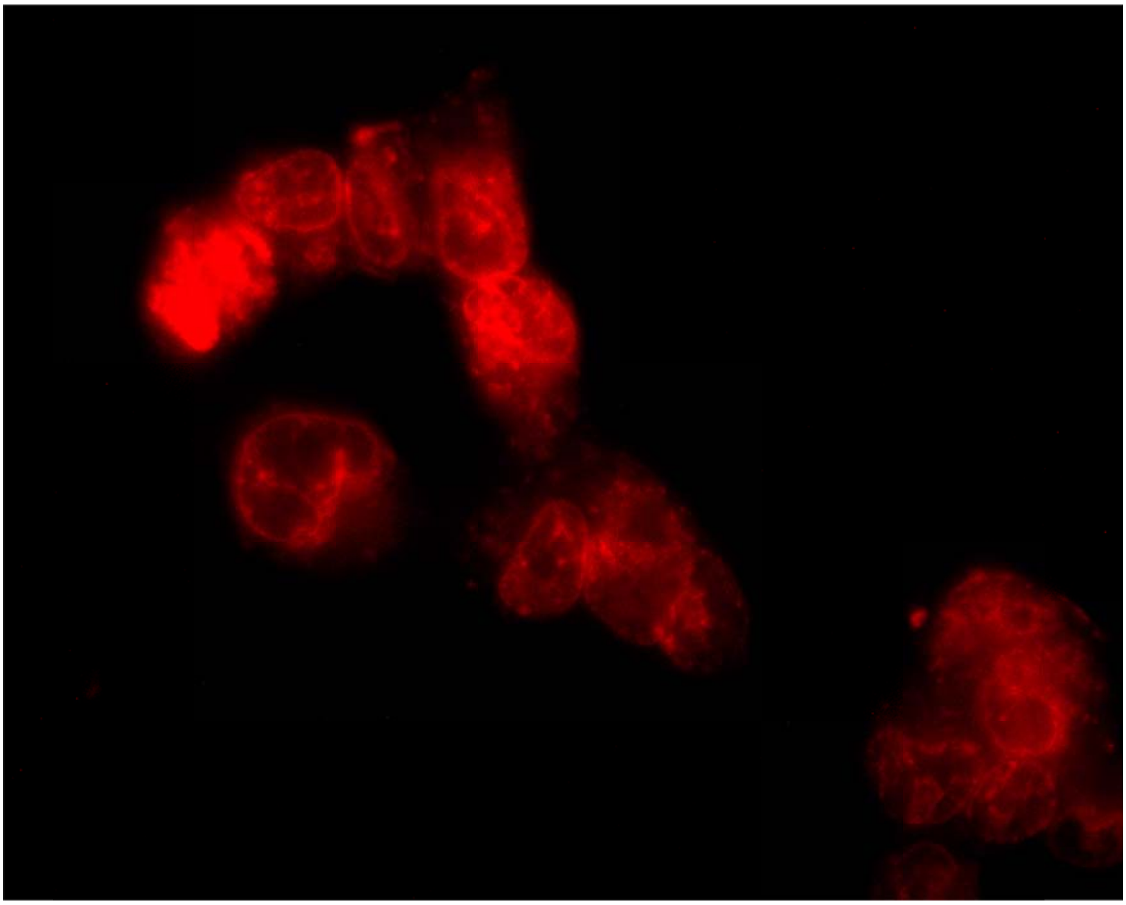

Supplemental Figure S5. High-resolution picture of doxorubicin accumulation fluorescence in Bafetinib (3 μM) treated HEK/ABCB1 cells.
